# Transarterial radioembolization vs transarterial chemoembolization with drug-eluting beads for treating hepatocellular carcinoma: a cost-effectiveness analysis in Japanese healthcare system

**DOI:** 10.1007/s11604-024-01640-1

**Published:** 2024-09-26

**Authors:** Go Shirota, So Sato, Hideo Yasunaga, Shotaro Aso, Masaaki Akahane, Daisuke Itoh, Osamu Abe

**Affiliations:** 1 Department of Radiology, Graduate School of Medicine, The University of Tokyo, 7-3-1 Hongo, Bunkyo-Ku, Tokyo, 113-8655 Japan; 2 Department of Clinical Epidemiology and Health Economics, School of Public Health, The University of Tokyo, Tokyo, Japan; 3 Department of Real-World Evidence, Graduate School of Medicine, The University of Tokyo, Tokyo, Japan; 4 Department of Radiology, International University of Health and Welfare Narita Hospital, Narita, Japan; 5 Department of Diagnostic Radiology, Toranomon Hospital, Tokyo, Japan

**Keywords:** Cost-effectiveness analysis, Hepatocellular carcinoma, Interventional radiology, Transarterial embolization, Yttrium-90

## Abstract

**Purpose:**

Transarterial radioembolization (TARE) is effective for unresectable hepatocellular carcinoma; however, it awaits approval in Japan. This study aimed to simulate the cost-effectiveness of TARE over chemoembolization when TARE is approved in Japan and identify the requirements for cost-effectiveness.

**Materials and methods:**

A Markov model was constructed to analyze the costs and effectiveness associated with TARE and transarterial chemoembolization with drug-eluting beads (DEB-TACE) for 2-month cycles over 5 years. In the primary analysis, the intention-to-treat survival data were used to calculate transition probabilities, whereas the ancillary analysis assessed the per-protocol survival data. DEB-TACE costs were calculated using the Japanese nationwide claims Diagnosis Procedure Combination database between April 2018 and March 2022, whereas TARE costs were estimated using database and international sources. The incremental cost-effectiveness ratio (ICER) was determined based on the payer’s perspective and compared with the Japanese willingness-to-pay threshold of 5 million Japanese yen (JPY) (31,250 USD) per quality-adjusted life years (QALY).

**Results:**

From the claims database, 6,986 patients with hepatocellular carcinoma who received DEB-TACE were identified. In the primary analysis, the ICER was 5,173,591 JPY (32,334 USD)/QALY, surpassing the Japanese willingness-to-pay threshold. However, the ancillary analysis showed a lower ICER of 4,156,533 JPY (25,978 USD)/QALY, falling below the threshold. The one-way deterministic sensitivity analysis identified progression-free survival associated with TARE and DEB-TACE, DEB-TACE costs, and radioactive microsphere reimbursement price as key ICER influencers. The primary analysis suggested that setting the reimbursement price of radioactive microspheres below 1.399 million JPY (8,744 USD), approximately 2.8% lower than the price in the United Kingdom, would place the ICER below the Japanese willingness-to-pay threshold.

**Conclusions:**

Under specific conditions, TARE can be a more cost-effective treatment than DEB-TACE. If the reimbursement price of radioactive microspheres is set approximately 2.8% lower than that in the United Kingdom, TARE could be cost-effective compared with DEB-TACE.

## Introduction

The decline in the incidence of viral hepatitis over the past decade has steadily decreased the age-standardized incidence and mortality rates of hepatocellular carcinoma (HCC) in Japan [1]. However, there is an increased per-patient medical cost associated with HCC [2]. This upward trend can be attributed to several factors, including the increased adoption of liver transplantation and the introduction of treatment regimens incorporating expensive drugs such as molecular-targeted agents and immune checkpoint inhibitors. HCC treatment is largely determined by the disease stage, as recommended by the Barcelona Clinic Liver Cancer (BCLC) staging system [3, 4] and especially in Japan, by the treatment algorithm on Clinical Practice Guidelines for Hepatocellular Carcinoma [5]. Transarterial therapies have played crucial roles in managing unresectable HCC, either by acting as a bridge to liver transplantation or by delaying the initiation of treatment regimens that include expensive drugs. The latter role is significant in Japan, where liver transplantation for HCC is primarily limited to cases classified as Child–Pugh class C [5].

Transarterial radioembolization (TARE) employing radioactive microspheres loaded with yttrium-90 (^90^Y), a pure beta-emitting isotope, is an alternative for treating unresectable HCC, potentially reducing treatment sessions with superior effectiveness compared with transarterial chemoembolization (TACE) [6]. Recent meta-analyses [7–9] detail the benefits of TARE over transarterial chemoembolization in terms of improved time-to-progression and survival. According to the 2022 update of the BCLC strategy for HCC, TARE could be considered for patients with BCLC-0 (very-early stage) and BCLC-A (early stage) with single nodules ≤ 8 cm, and patients with BCLC-C (advanced stage). TARE had also been suggested to be as effective as sorafenib in patients with liver-only involvement [4]. In Japan, TARE has not yet received health insurance approval, and the Japanese Society of Interventional Radiology is currently requesting approval [10]. However, the substantial cost associated with TARE is a significant challenge in healthcare economics. Radioactive microspheres for TARE cost 8,000 GBP in the United Kingdom [11], which is approximately 14 times more expensive than the reimbursement price of drug-eluting beads and chemotherapeutic agents for transarterial chemoembolization with drug-eluting beads (DEB-TACE) in Japan [12]. Even if TARE requires fewer treatment sessions and provides better effectiveness than chemoembolization, it should be cost-effective to be covered by public health insurance. In Japan, because of the increase in extremely expensive treatments, cost-effectiveness analysis was introduced in 2019 to determine the pricing of ultra-high-cost drugs and devices [13].

There have been no previous cost-effectiveness studies for TARE based on the unique characteristics of Japan because previous studies in Europe and the United States primarily focused on models based on bridging to transplantation [11, 14–16]. In Japan, transarterial therapy plays a role in delaying the initiation of expensive systemic pharmacotherapy, and liver transplantation for HCC is primarily limited to cases classified as Child–Pugh class C [5]. Therefore, cost-effectiveness analyses comparing TARE and chemoembolization must be based on a comprehensive and lifetime model that considers the costs and effectiveness of systemic pharmacotherapy after patients become refractory to transarterial therapies.

This study addresses this challenge by conducting a comprehensive cost-effectiveness analysis of TARE in comparison to DEB-TACE. Historically, in Japan conventional TACE (cTACE, lipiodol TACE) was developed as standard therapy for unresectable HCC, and DEB-TACE was not covered by Japanese insurance until 2014. There has been much debate on the indications for cTACE and DEB-TACE. An international randomized controlled trial (RCT) conducted in Europe (PRECISION V study) reported that DEB-TACE is a safe and effective treatment of HCC and is beneficial for patients with more advanced disease [17]. Another RCT conducted in Italy revealed that DEB-TACE and TACE are equally effective and safe, and less post-procedural abdominal pain is an advantage of the former [18]. A recent RCT in performed Japan revealed that selective cTACE appeared to yield higher complete response rates for local tumor control compared to selective DEB-TACE for HCC. However, the frequency of postembolization syndrome was also significantly higher in the cTACE group than in the DEB-TACE group [19]. Thus, the current consensus is that, while cTACE is more effective than DEB-TACE in patients with small and confined tumors, DEB-TACE is preferable in patients with a higher tumor burden and poor liver function facing higher risk of postembolization syndrome. Because TARE is recommended for large HCC [20], it will likely be indicated for patients with a higher tumor burden when it is approved in Japan. Therefore, our study compared the cost-effectiveness of TARE versus DEB-TACE instead of cTACE.

Our model relies on the published clinical outcomes of studies, including an RCT [6], and real-world data extracted from the Diagnosis Procedure Combination (DPC) database [21], a Japanese nationwide administrative claims database. Through this analysis, we aimed to contribute to the ongoing discourse surrounding insurance coverage for TARE and describe the economic implications of this innovative approach to HCC management. Specifically, this study aimed to guide the appropriate reimbursement pricing policy for TARE procedures and radioactive microspheres from the perspective of cost-effectiveness in the Japanese healthcare system.

## Materials and methods

This study was approved by the Ethics Committee of the University of Tokyo (No. 3501-(5)). Because all data obtained from the DPC database were anonymized, the requirement for patient informed consent was waived.

### Model

**Fig. 1:**
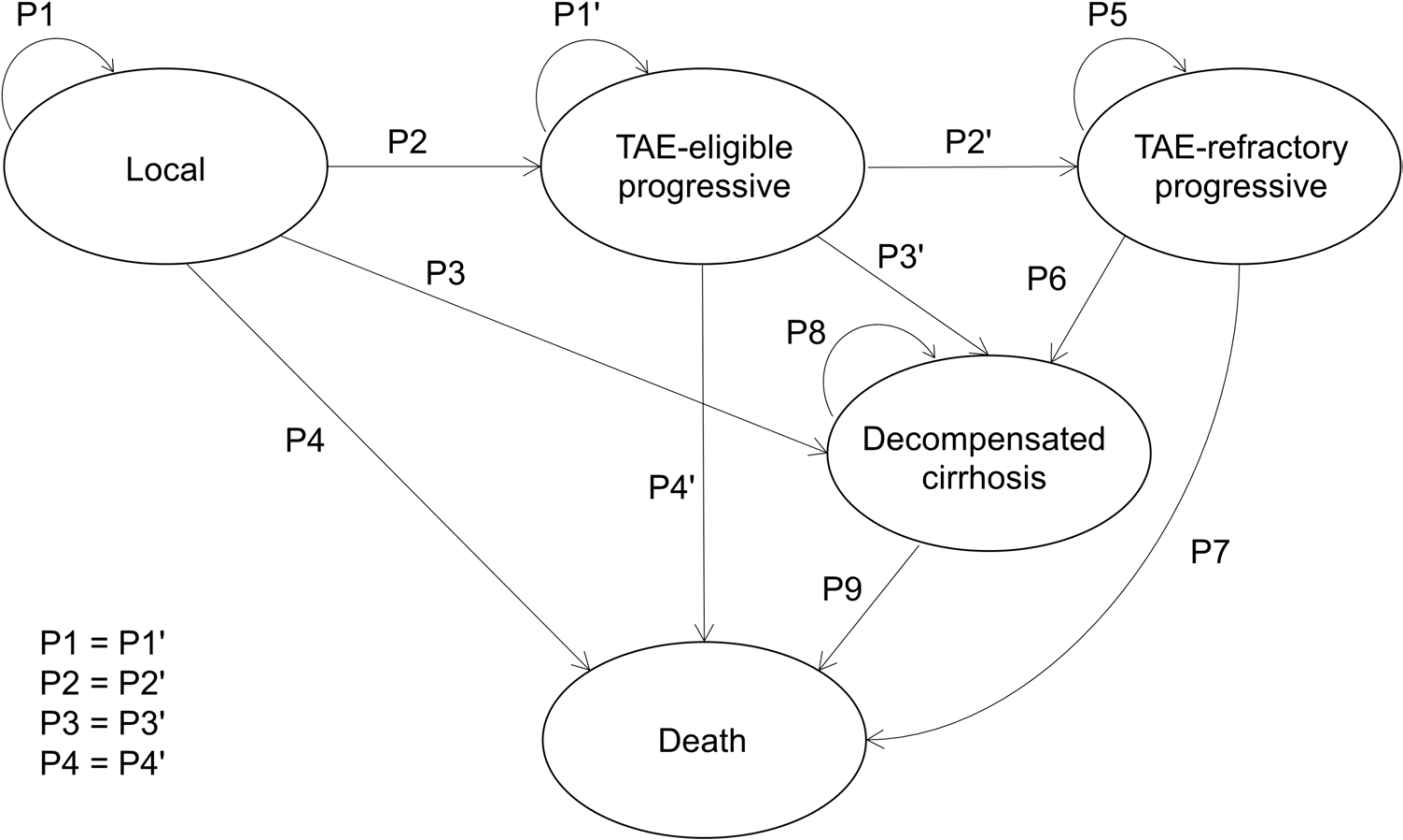
A Markov Model with five health states: local, transarterial embolization (TAE)-eligible progressive, TAE-refractory progressive, decompensated cirrhosis, and death was constructed. Arrows indicate state transitions. Patients started in the local state and received TARE or DEB-TACE within the first cycle. Subsequently, patients remained in the local state, transitioned to the TAE-eligible progressive state, developed non-compensated cirrhosis, or died based on transition probabilities. Patients who progressed further after two TARE or DEB-TACE sessions were ineligible for additional sessions and transitioned to a TAE-refractory progressive state. *DEB-TACE* transarterial chemoembolization with drug-eluting beads, *TAE* transarterial embolization, *TARE* transarterial radioembolization

We developed a Markov model to compare the cost-effectiveness of TARE and DEB-TACE for HCC. Markov processes can simulate the long-term health states of a patient by tracking the patient's transitions between the health states defined in the model. During each cycle, patients transition according to the transition probabilities and accumulate the quality-adjusted life year (QALY) and cost for each state. Our model spans 5 years of 2-month cycles based on previous studies [6, 14, 15], with five health states: local, transarterial embolization (TAE)-eligible progressive, TAE-refractory progressive, decompensated cirrhosis, and death (Fig. 1). Our hypothetical cohort included patients with unresectable HCC who were not eligible for percutaneous ablation, partial hepatectomy, or transplantation based on the inclusion criteria set by an RCT comparing TARE and DEB-TACE [6]. Patients began in the local state and received TARE or DEB-TACE within the first cycle. Subsequently, patients remained in the local state, transitioned to the TAE-eligible progressive state, developed non-compensated cirrhosis, or died based on transition probabilities. Patients in the TAE-eligible progressive state were still eligible for TARE or DEB-TACE. Patients who progressed further after two TARE or DEB-TACE sessions were ineligible for additional sessions and transitioned to a TAE-refractory progressive state according to the definition of “TACE failure” in the Japanese Clinical Practice Guidelines for HCC [5]. Patients in the TAE-refractory progressive state, following the algorithm for drug therapy in the Japanese guideline, were recommended to receive treatment regimens, including immune checkpoint inhibitors (atezolizumab + bevacizumab). In recent years, in actual clinical practice, systemic therapy is often started before chemoembolization, and then, chemoembolization is performed on demand. However, in this study, to simplify the model and to conform to the Japanese guideline, we used a model in which chemoembolization was performed first, and systemic therapy was performed only in patients who became TAE refractory. The total maximum sessions for DEB-TACE and TARE were set at six and two, respectively, based on previous studies [6, 14]. Patients in other health states transitioned to decompensated cirrhosis, indicating the need for palliative care. Costs and outcomes were discounted at 2% annually, following the Japanese guideline for economic evaluation of drugs and medical devices [22].

### Transition probabilities

Transition probabilities were calculated using survival and decompensation rate data from previous studies [6, 23–26]. In the primary analysis, we used survival data from the intention-to-treat analysis of an RCT that compared TARE and DEB-TACE [6] to calculate transition probabilities, whereas we used survival data from the per-protocol analysis in the ancillary analysis. Transition probabilities were calculated using a method similar to a previous cost-effectiveness analysis of small-cell lung cancer treatment [27]. Based on the assumption that the survival curves of previous trials can be approximated by an exponential function, the transition probabilities were calculated using the following formula based on the previous study, which provides the equations: P (transition probabilities) = 1 − (0.5)^(length of cycle/median time to event)^ [27]. Our model also considers the development of decompensated cirrhosis with certain transition probabilities based on previous cohort studies [24, 25]. As shown in Fig. 1, the transition probability from local to local equals that of TAE-eligible progressive to TAE-eligible progressive (P1 = P1'); transition probability from local to TAE-eligible progressive equals that of TAE-eligible progressive to TAE-refractory progressive (P2 = P2'); the transition probability from local to decompensated cirrhosis equals that of TAE-eligible progressive to decompensated cirrhosis (P3 = P3'), and the transition probability from local to death equals that of TAE-eligible progressive to death (P4 = P4') based on the assumption that survival curves that approximate the same exponential function are applied in states eligible for TARE or DEB-TACE. Appendix E1 provides detailed calculation formulas.

### Utilities

The utility data were estimated from quality-of-life scores based on the EuroQol 5 dimensions scale from a previous study on HCC and hepatitis C virus-related diseases in Japan [28] and another study evaluating the cost-effectiveness of lenvatinib versus sorafenib for the treatment of patients with unresectable HCC in Japan using data from the REFLECT Trial [29]. The quality-of-life scores of the “local state” and “TAE-refractory progressive state” were derived from the mean of “HCC-intermediate” [28] and “progression-free” [29], and the mean of “HCC-advanced” [28] and”progressed” [29], respectively, reported by previous studies. The quality-of-life score of the “TAE-eligible progressive state” is estimated based on the assumption that it is the mean of the score of the “local state” and “TAE-refractory progressive state.”

### Costs

The costs of DEB-TACE were calculated using the DPC database [21], a Japanese nationwide inpatient database that includes hospital administrative claims data and discharge abstracts for 8 million inpatients in over 1,200 hospitals throughout Japan. It includes data recorded during hospitalization (sex, age, diagnosis, surgical procedure, International Classification of Diseases 10th Revision [ICD-10] codes, and total hospitalization cost). This database was used to identify patients with HCC treated using DEB-TACE between April 2018 and March 2022. The following search criteria were used to select patients from the database: (1) claiming selective TACE (Japanese procedure claiming code; K615_2) as the procedure fee; (2) HCC (ICD-10 code: C220) was the major disease requiring hospitalization; (3) claiming drug-eluting beads; and (4) not claiming lipiodol (excluding conventional TACE). Hospitalization costs for the following two patient cohorts were considered: patients solely reimbursed for TACE (without severe hepatobiliary complications) and those with severe hepatobiliary complications. Patients with severe hepatobiliary complications were defined as those who claimed reimbursement for the following procedures after DEB-TACE: percutaneous gallbladder or liver abscess drainage (claim code: J010-2), surgical biliary drainage (claim codes: K682, K696, K697), percutaneous transhepatic biliary drainage (claim code: K682-2), endoscopic biliary drainage (claim codes: K682-3), endoscopic biliary stent (claim code: K688), percutaneous transhepatic biliary stent (claim code: K689), surgical liver abscess drainage (claim code: K691), percutaneous liver abscess drainage (claim code: K691-2), and surgical liver abscess resection (claim code: K694). The number of patients with severe hepatobiliary complications and the cost of hospitalization for these patients were extracted from the DPC database. The cost of potential complications considering the occurrence of severe hepatobiliary complications was estimated. The weighted average of these costs represented the average hospitalization cost for DEB-TACE applied to the local and TAE-eligible progressive states.

The costs of TARE were estimated differently because TARE lacks approval from Japanese health insurance companies. The costs of DEB-TACE were categorized into components assuming that the shared costs between TARE and DEB-TACE, such as procedure fees, reimbursable devices (sheath, catheter, microcatheter, and guidewire), daily hospitalization costs, and potential complications, would be identical. In some countries, TARE is performed as an outpatient procedure; however, we assumed that it would be performed as an admission procedure in Japan. Costs unique to TARE, such as pre-procedure angiography, scintigraphy, and cost of radioactive microspheres, were considered additional expenses. The costs of pre-procedural angiography and scintigraphy were added only once for the first TARE session. The costs of pre-procedure angiography, scintigraphy, and contrast computed tomography for follow-up in the local and TAE-eligible progressive states were determined based on the national fee schedule. However, the cost of radioactive microspheres was derived from a study conducted in the United Kingdom [11]. To estimate the potential costs for early severe complications, we assumed that there was no significant difference in the risk of severe complications between TARE and DEB-TACE according to the results of a previous RCT that found no significant differences in the frequency of participants with at least one serious adverse event until 6 months after treatment and thirty-day mortality between the TARE and DEB-TACE arms [6].

The costs of treatment regimens involving immune checkpoint inhibitors (atezolizumab + bevacizumab) for the TAE-refractory progressive state were sourced from the White Paper published by the Japan Society of Hepatology [30]. Costs for decompensated cirrhosis and outpatient therapy for the local and TAE-eligible progressive states were extrapolated from previous Japanese studies using real-world claim data for viral hepatitis-related diseases and nonalcoholic fatty liver disease [31–33].

### Cost-effectiveness analysis

The current study adhered to the Consolidated Health Economic Evaluation Reporting Standards 2022 Statement and Japanese guidelines [22, 34, 35]. The perspective considered was that of healthcare payers under the Japanese system, including only the monetary costs directly attributed to disease management and excluding indirect costs such as productivity loss caused by the disease [36]. The incremental cost-effectiveness ratio (ICER) was calculated as the incremental cost per QALY of TARE compared with that of DEB-TACE and compared with a benchmark for the willingness-to-pay [5 million Japanese yen (JPY) per QALY [37]](31,250 USD/QALY). The ICER must be smaller than the willingness-to-pay threshold for the treatment to be considered cost-effective. In other words, TARE would be considered socially acceptable if the incremental cost to gain one incremental QALY compared to DEB-TACE is within the willingness-to-pay threshold (5 million JPY). The costs and ICER are also expressed in US dollars using a currency exchange rate of JPY 160 per 1 US dollar.

One-way deterministic sensitivity analysis was conducted to identify input parameters that exerted the most substantial influence on ICER. Cost and utility inputs were subjected to a deviation of 10%, whereas survival data employed in generating transition probabilities were subjected to a deviation of 20–30% according to the original research’s standard deviation. The discount rate range was determined as 0–4% according to the Japanese guideline [22].

Furthermore, probabilistic sensitivity analysis (Monte Carlo simulation) was performed to simulate 10,000 patients in the TARE and DEB-TACE cohorts. Based on previous research, probability and utility inputs followed beta distributions with a standardized difference of 10%, whereas cost inputs followed gamma distributions with a similar standardized difference [11]. The results of these simulations generated acceptability curves, illustrating the probability of cost-effectiveness at different willingness-to-pay thresholds.

Analyses were conducted using Stata/MP 16.0 (StataCorp, College Station, TX, USA) and TreeAge Pro Healthcare 2023 (TreeAge Software, Inc., Williamstown, MA, USA).

## Results

### Transition probabilities, utilities, and costs

**Fig. 2:**
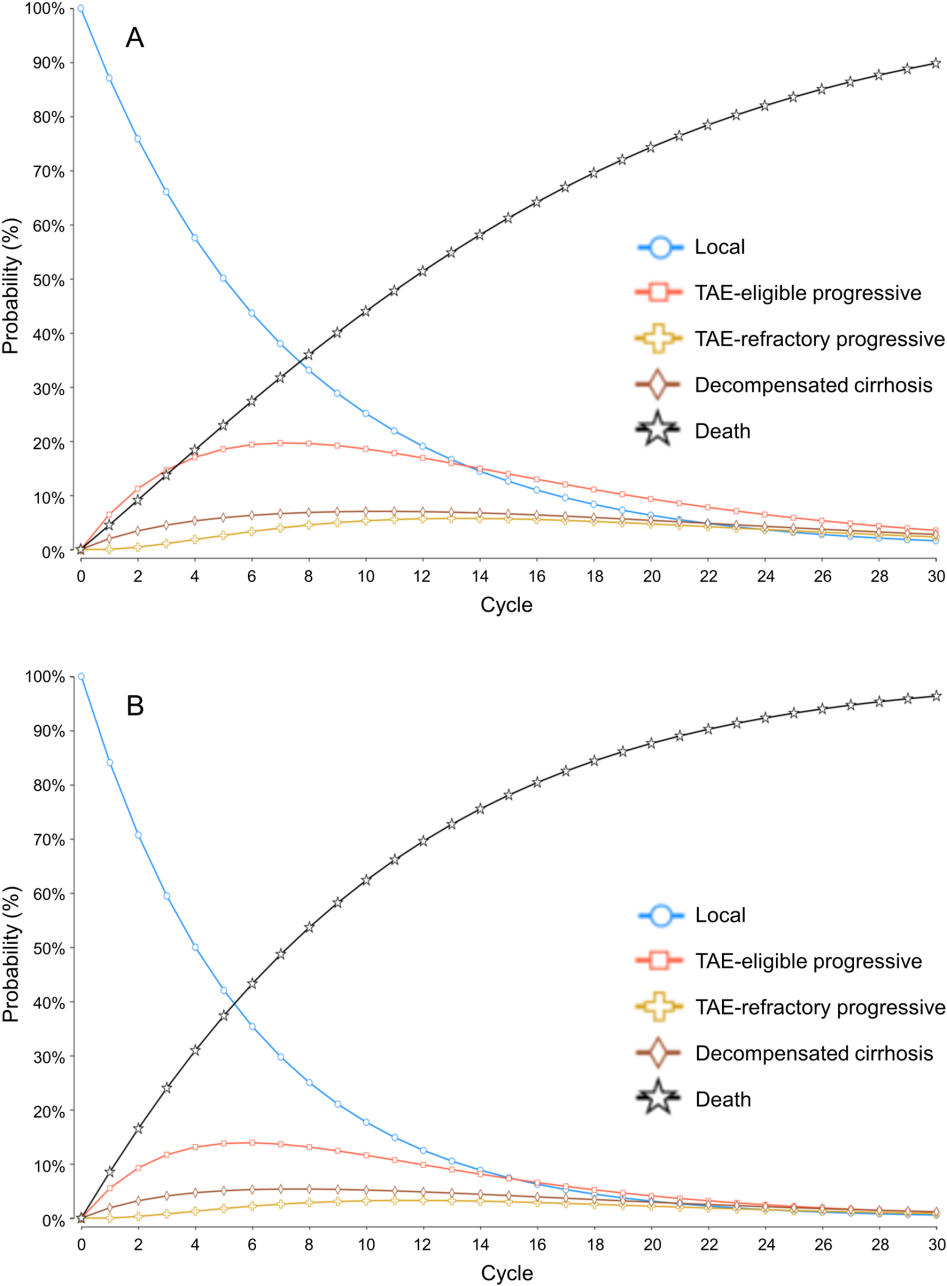
State probability graph of TARE (**A**) and DEB-TACE (**B**) (Primary Analysis). The probabilities of each state per cycle are plotted for 30 cycles (= 5 years). DEB-TACE, transarterial chemoembolization with drug-eluting beads; *TAE* transarterial embolization, *TARE* transarterial radioembolization

Tables 1 and 2 list the baseline parameters, including the utilities and costs in the model and transition probabilities, respectively. Figure 2 shows the projected survival curves based on these estimated probabilities.

**Table 1:** Baseline parameters used in the model

Variable	Mean value (range)	Distribution	Reference
*Cost (JPY)*			
DEB-TACE (total cost per administration)	845,320 (± 20%)	Gamma (SD 10%)	DPC data
Reimbursement price of radioactive microspheres	1,440,000 (± 20%)	Gamma (SD 10%)	Cost of TheraSphere ™ in the United Kingdom [11]
TARE (cost per administration other than radioactive microspheres)	734,896 (± 20%)	Gamma (SD 10%)	Estimated from DPC data of DEB-TACE Details on Table 3
Outpatient therapy (monthly)	98,455 (± 20%)	Gamma (SD 10%)	Estimated from previous studies [31–33]
Progressive state (monthly)	1,242,800 (± 20%)	Gamma (SD 10%)	Estimated from Japanese White Paper on Liver Cancer [30]
Decompensated cirrhosis (monthly)	247,690 (± 20%)	Gamma (SD 10%)	Estimated from previous studies [31–33]
†Pre-procedural angiography for TARE	36,000	Gamma (SD 10%)	National fee schedule of Japan 2022
†Scintigraphy for TARE	22,215	Gamma (SD 10%)	National fee schedule of Japan 2022
Contrast-enhanced CT	30,000	Gamma (SD 10%)	National fee schedule of Japan 2022
Utility (quality-of-life scores)			
Local state	0.788 (± 10%)	Beta (SD 10%)	‡ Derived from previous study [28, 29]
TAE-eligible progressive state	0.768 (± 10%)	Beta (SD 10%)	‡ Derived from previous study [28, 29]
TAE-refractory progressive state	0.748 (± 10%)	Beta (SD 10%)	‡ Derived from previous study [28, 29]
Decompensated cirrhosis	0.524 (± 10%)	Beta (SD 10%)	Derived from previous study [28]
*Factors used for calculating transition probabilities*			
Median OS of DEB-TACE (month)	ITT 15.6 (± 30%) PP 15.6 (± 30%)	Gamma (SD 10%)	Derived from RCT [6]
Median OS of TARE (month)	ITT 30.2 (± 30%) PP 30.2 (± 30%)	Gamma (SD 10%)	Derived from RCT [6]
Median PFS of DEB-TACE (month)	ITT 9.10 (± 30%) PP 9.10 (± 30%)	Gamma (SD 10%)	Derived from RCT [6]
Median PFS of TARE (month)	ITT 11.83 (± 30%) PP 12.83 (± 30%)	Gamma (SD 10%)	Derived from RCT [6]
Median OS of atezolizumab + bevacizumab therapy (month)	19.2 (± 30%)	Gamma (SD 10%)	Derived from RCT [23]
Annual decompensation rate of TAE-eligible population (%)	11.8 (± 30%)	Beta (SD 10%)	Derived from previous cohort study [24]
Annual decompensation of atezolizumab + bevacizumab therapy (%)	53.4 (± 30%)	Beta (SD 10%)	Derived from previous real-world cohort [25]
Annual survival rate of decompensated cirrhosis (%)	25.0 (± 30%)	Beta (SD 10%)	Derived from previous cohort study [26]
Maximum sessions of DEB-TACE	6	NA	Assumption based on previous studies [6, 14]
Maximum sessions of TARE	2	NA	Assumption based on previous studies [6, 14]
Length of cycle (month)	2	NA	Assumption based on previous studies [6, 14]
Total cycles	30	NA	Assumption based on previous studies [14, 15]
Discount rate (%, yearly)	2	Triangular (0–4)	Derived from Japanese guideline [35]

*Range was used for one-way deterministic sensitivity analysis, whereas distribution was used for probabilistic sensitivity analysis

^†^The cost of pre-procedure angiography and scintigraphy was added only to the cost of the first session of TARE

^‡^The quality-of-life score of “Local state” and “TAE-refractory progressive state” were derived from the mean of “HCC-intermediate” [28] and “progression-free” [29] and the mean of “HCC-advanced” [28] and “progressed” [29] in the previous studies, respectively. The quality-of-life score of the “TAE-eligible progressive state” is estimated based on the assumption that it is the mean of the score of the “Local state” and “TAE-refractory progressive state.”

*DEB-TACE* transarterial chemoembolization with drug-eluting beads, *DPC* diagnostic procedure combination, *ICER* incremental cost-effectiveness ratio, *ITT* intention-to-treat, *JPY* Japanese yen, *NA* not available, *OS* overall survival, *PFS* progression-free survival, *PP* per-protocol, *RCT* randomized controlled trial, *SD* standard deviation, *TAE* transarterial embolization, *TARE* transarterial radioembolization

**Table 2:** Transition Probabilities (%)

State Transition	TARE	DEB-TACE	Source
Local to local (P1)	87.10 (ITT) 87.90 (PP)	84.10 (ITT) 84.10 (PP)	Derived using OS/PFS data from Dhondt et al.[6] and annual decompensation data from Fleming et al.[24]
TAE-eligible progressive to TAE-eligible progressive (P1')	87.10 (ITT) 87.90 (PP)	84.10 (ITT) 84.10 (PP)	Based on the assumption that equals the transition probability from local to local (P1 = P1')
Local to TAE-eligible progressive (P2)	6.44 (ITT) 5.64 (PP)	5.51 (ITT) 5.51 (PP)	Derived using OS/PFS data from Dhondt et al.[6] and annual decompensation data from Fleming et al.[24]
TAE-eligible progressive to TAE-refractory progressive (P2')	6.44 (ITT) 5.64 (PP)	5.51 (ITT) 5.51 (PP)	Based on the assumption that equals the transition probability from Local to TAE-eligible progressive (P2 = P2')
Local to decompensated cirrhosis (P3)	1.98 (ITT) 1.98 (PP)	1.89 (ITT) 1.89 (PP)	Derived using OS/PFS data from Dhondt et al.[6] and annual decompensation data from Fleming et al.[24]
TAE-eligible progressive to decompensated cirrhosis (P3')	1.98 (ITT) 1.98 (PP)	1.89 (ITT) 1.89 (PP)	Based on the assumption that equals the transition probability from local to decompensated cirrhosis (P3 = P3')
Local to death (P4)	4.49 (ITT) 4.49 (PP)	8.50 (ITT) 8.50 (PP)	Derived using OS/PFS data from Dhondt et al.[6] and annual decompensation data from Fleming et al.[24]
TAE-eligible progressive to death (P4')	4.49 (ITT) 4.49 (PP)	8.50 (ITT) 8.50 (PP)	Based on the assumption that equals the transition probability from local to death (P4 = P4')
TAE-refractory progressive to TAE-refractory progressive (P5)	81.92 (ITT) 81.92 (PP)	81.94 (ITT) 81.94 (PP)	Derived using OS data from Cheng et al.[23] and annual decompensation data from Jost-Brinkmann et al.[25]
TAE-refractory progressive to decompensated cirrhosis (P6)	11.1	11.1	Derived using OS data from Cheng et al.[23] and annual decompensation data from Jost-Brinkmann et al.[25]
TAE-refractory progressive to death (P7)	6.97	6.97	Derived using OS data from Cheng et al.[23] and annual decompensation data from Jost-Brinkmann et al.[25]
Decompensated cirrhosis to decompensated cirrhosis (P8)	79.4	79.4	Derived using 1 year survival data from Kudo et al.[26]
Decompensated cirrhosis to death (P9)	20.6	20.6	Derived using 1 year survival data from Kudo et al.[26]

Note—Details of the calculation formulas for the transition probabilities are provided in the Supplementary Data (Appendix E1). The probabilities represented by P1 to P9 each correspond to the probabilities in the schema of the Markov model in Fig. 1

*DEB-TACE* transarterial chemoembolization with drug-eluting beads, *ITT* intention-to-treat, *OS* overall survival, *PFS* progression-free survival, *PP* per-protocol, *TAE* transarterial embolization, *TARE* transarterial radioembolization

From the DPC database, we identified 6,986 patients (74.86 ± 9.67 years old; 5196 males, 1,790 females; 6961 without any complication requiring interventional procedures, 25 patients with severe hepatobiliary complications requiring interventional procedures) with HCC treated with DEB-TACE between April 2018 and March 2022. The age of patients identified from the DPC database was comparable to the mean age at clinical diagnosis of HCC in Japan (70.0 and 74.0 years for men and women, respectively [38]) and tended to be higher than that of patients in the RCT used to calculate the transition probability (median age: 67 years for the TARE arm and 68 years for the DEB-TACE arm [6]). Based on the DPC data, the mean cost of hospitalization for DEB-TACE was 839,223 JPY (5245 USD) without complications and 2,543,046 JPY (15,894 USD) with severe hepatobiliary complications. The cost of potential complications considering the occurrence of severe hepatobiliary complications was 6097 JPY (38 USD). Based on these data, the weighted average cost of DEB-TACE was estimated as 845,320 JPY (5,283 USD).

Using these data, the cost of hospitalization for TARE (costs other than radioactive microspheres) was estimated to be 734,896 JPY (4,593 USD). The cost of radioactive microspheres was calculated as 1,440,000 JPY (9000 USD)[11], and the total cost of hospitalization for TARE was estimated as 2,174,896 JPY (13,593 USD). Table 3 lists the detailed cost estimations for TARE. These data were input into the model as the costs of the local and TAE-eligible progressive states.

**Table 3:** Estimation of Costs for TARE (JPY)

Cost Item	TARE	DEB-TACE	Reference
Procedure fee	200,400	200,400	Procedure fee of TACE on the National fee schedule of Japan 2022
*Reimbursement for medical devices and drugs*			
Sheath, catheter, guidewire, microcatheter, micro-guidewire	71,800	71,800	List price on the National fee schedule of Japan 2022
Drug-eluting beads	NA	103,000	List price of a vial of DC Bead™ and HepaSphere™ on the national fee schedule of Japan 2022
Chemotherapeutic agents	NA	7,424	List price of a vial of epirubicin 50 mg on the national fee schedule of Japan 2022
Per-diem payment (hospitalization costs including meals, drugs other than those used in catheterization laboratory)	456,599	456,599	DPC data
Cost of potential complications	6,097	6,097	DPC data (considering the costs and the occurrence of severe hepatobiliary complications)
Subtotal of costs other than radioactive microspheres	734,896 (A)		
Radioactive microspheres	1,440,000 (B)	NA	Price of Therasphere™ in the United Kingdom[11] converted at an exchange rate of 180 JPY/GBP
Total	2,174,896 (A + B)	845,320	

Note—* The costs of pre-procedural angiography and scintigraphy were added only to the cost of the first TARE session

^†^Based on the assumption that there is no significant difference in the risk of severe complications between TARE and DEB-TACE according to the result of a previous trial [6]

*DEB-TACE* transarterial chemoembolization with drug-eluting beads, *DPC* diagnostic procedure combination, *GBP* Great Britain pound, *JPY* Japanese yen, *NA* Not applicable, *TARE* transarterial radioembolization

### Cost-effectiveness analysis

Table 1 lists the cost and QALY of each therapy based on the Markov model. In the primary analysis, using the survival data from the intention-to-treat analysis result, the 5-year follow-up costs were estimated to be 7,499,735 JPY (46,873 USD) and 9,853,942 JPY (61,587 USD) for DEB-TACE and TARE, respectively. The incremental QALYs were estimated as 1.22 years and 1.68 years for DEB-TACE and TARE, respectively. The ICER was 5,173,591 JPY/QALY (32,335 USD/QALY), which exceeded the benchmark of willingness-to-pay (5 million JPY/QALY [37]). Using the survival data of the per-protocol analysis result in the ancillary analysis, the ICER was 4,156,533 JPY/QALY (25,978 USD/QALY), below the willingness-to-pay benchmark. These data are summarized in Table 4.

**Table 4:** Cost-effectiveness of TARE vs. DEB-TACE

	TARE	DEB-TACE	Incremental Value
*Cost (JPY)*			
Primary analysis (ITT)	9,853,942 (61,587 USD)	7,499,735 (46,873 USD)	2,354,206 (14,714 USD)
Ancillary analysis (PP)	9,474,170 (59,214 USD)	7,499,735 (46,873 USD)	1,974,434 (12,340 USD)
*Effectiveness (QALY)*			
Primary analysis (ITT)	1.68	1.22	0.46
Ancillary analysis (PP)	1.70	1.22	0.48
*ICER (JPY/QALY)*			
Primary analysis (ITT)			5,173,591 (32,335 USD/QALY)
Ancillary analysis (PP)			4,156,533 (25,978 USD/QALY)

*DEB-TACE* transarterial chemoembolization with drug-eluting beads, *ICER* incremental cost-effectiveness ratio, *JPY* Japanese yen, *ITT* intention-to-treat, *PP* per-protocol, *QALY* quality-adjusted life year, *TARE* transarterial radioembolization

**Fig. 3:**
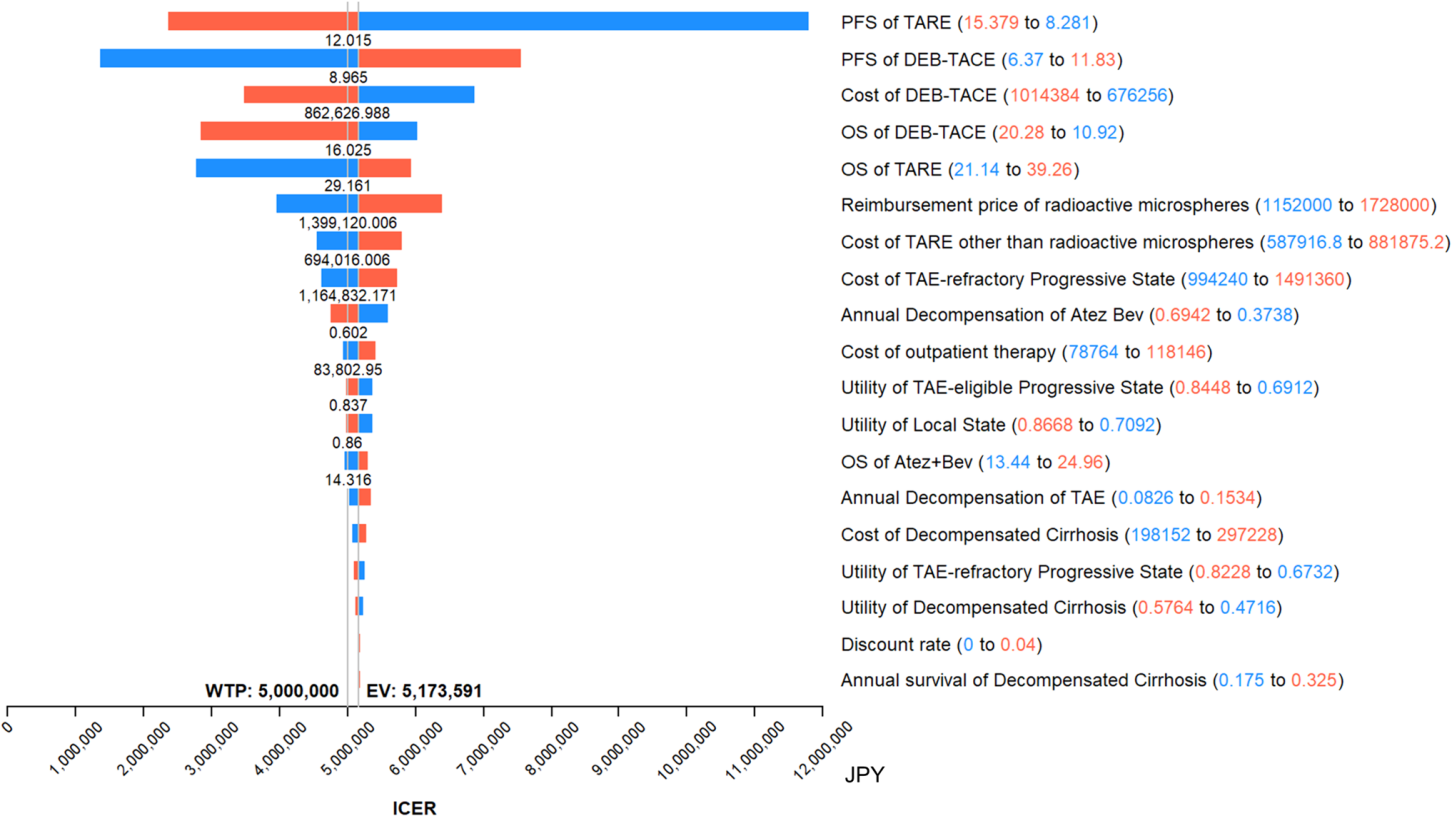
One-way deterministic sensitivity analysis results as a tornado diagram in the primary analysis. Upper parameters have a greater impact on the incremental cost-effectiveness ratio. The red bar shows the change when the parameter is increased, and the blue bar shows the change when the parameter is decreased. The numbers below the bars of some parameters indicate the threshold variable value, indicating the threshold below which the incremental cost-effectiveness ratio falls below the willingness-to-pay threshold if the parameter is above or below this value. *Atez + Bev* atezolizumab + bevacizumab, *DEB-TACE* transarterial chemoembolization with drug-eluting beads, *EV* expected value, *ICER* incremental cost-effectiveness ratio, *JPY* Japanese yen; OS, overall survival; PFS, progression-free survival, *TAE* transarterial embolization, *TARE* transarterial radioembolization, *WTP* willingness-to-pay

Figure 3 shows the results of the one-way deterministic sensitivity analysis (performed in the primary analysis) as a tornado diagram. The parameter with the greatest influence on the ICER was the median PFS, which was used to calculate the transition probabilities from the local state to the TAE-eligible progressive state and from the TAE-eligible progressive state to the TAE-refractory progressive state. The one-way deterministic sensitivity analysis in the primary analysis suggested that reducing the reimbursement price of radioactive microspheres from 1.440 million JPY (9,000 USD) to 1.399 million JPY (8,745 USD), approximately 2.8% lower than the price in the United Kingdom, would align the ICER with the 5 million JPY/QALY threshold. In contrast, the impact of the utility of each state on the ICER was small.

**Fig. 4:**
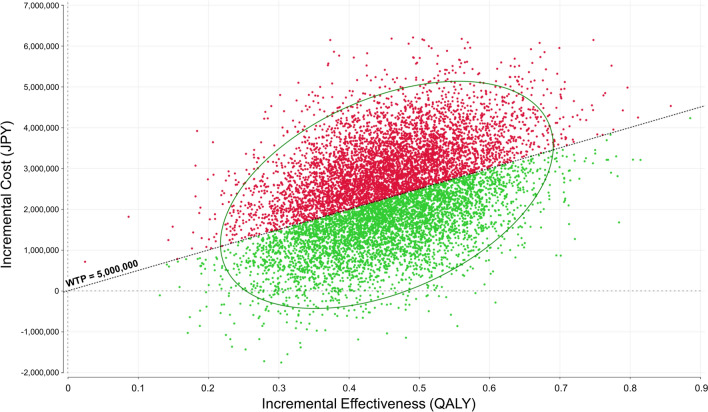
Probabilistic sensitivity analysis for the cost-effectiveness of TARE compared with that of DEB-TACE (Primary Analysis). The dots denote the results of Monte Carlo simulations of 10,000 samples. In the primary analysis, using the survival data of the intention-to-treat analysis result, less than half of the estimates in the probabilistic sensitivity analysis with Monte Carlo simulations were located below a line of willingness-to-pay of 5 million JPY/QALY. *TARE* transarterial radioembolization, *DEB-TACE* transarterial chemoembolization with drug-eluting beads, *JPY* Japanese yen, *QALY* quality-adjusted life year, *WTP* willingness-to-pay

**Fig. 5:**
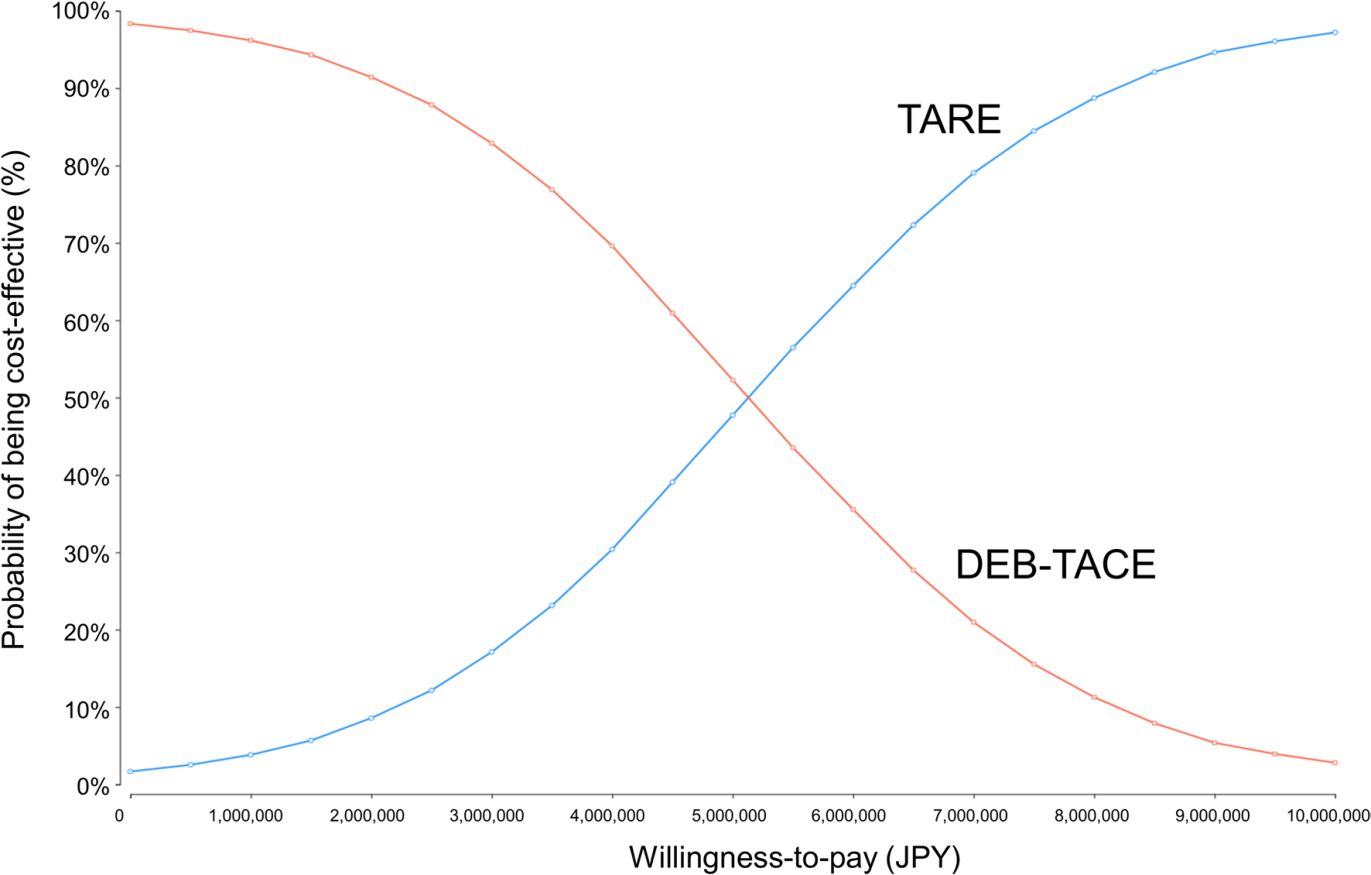
Acceptability curve based on the probabilistic sensitivity analysis of the cost-effectiveness of TARE compared with that of DEB-TACE (Primary Analysis). When the willingness-to-pay was approximately > 5.17 million JPY, TARE was likely to be more cost-effective than DEB-TACE. *DEB-TACE* transarterial chemoembolization with drug-eluting beads, *JPY* Japanese yen, *TARE* transarterial radioembolization

In the primary analysis, using the survival data of the intention-to-treat analysis result, less than half of the estimates in the probabilistic sensitivity analysis with Monte Carlo simulations were located below the willingness-to-pay benchmark of 5 million JPY/QALY (Fig. 4). Therefore, TARE was less likely to be cost-effective than DEB-TACE in the primary analysis. Figure 5 shows the acceptability curve based on probabilistic sensitivity analysis. In the primary analysis, when the willingness-to-pay was approximately more than 5.17 million JPY (approximately 32,300 USD), TARE was likely to be more cost-effective than DEB-TACE.

## Discussion

This study conducted a comprehensive cost-effectiveness analysis using a model tailored to Japanese clinical practice and real-world cost data. The primary analysis performed using the intention-to-treat survival data revealed that the ICER of TARE over DEB-TACE exceeded the Japanese willingness-to-pay threshold of 5 million JPY/QALY, reaching approximately 5.17 million JPY/QALY (32,300 USD/QALY). However, in the ancillary analysis, utilizing the per-protocol survival data, the ICER was approximately 4.16 million JPY/QALY (26,000 USD/QALY), falling below the willingness-to-pay threshold.

Since TARE is an expensive treatment, several North American and European studies have investigated its financial aspect. A systematic review included 20 economic evaluations (11 full economic evaluations and 9 partial economic evaluations) [39]. Out of 11 full economic evaluations, 4 studies compared TARE with TACE (two from the United States [14, 40], one from Italy [41] and one from the United Kingdom [11]) and 7 studies compared TARE with tyrosine kinase inhibitors. Transarterial therapies are commonly used for downstaging or bridging for liver transplantation in Europe and the United States; however, Japan restricts liver transplantation for HCC to Child–Pugh class C cases. Notably, previous cost-effectiveness studies in Europe and the United States primarily focused on models based on bridging transplantation [11, 14–16]. Therefore, our model is applicable in countries where liver transplantation is not widely used for unresectable HCC. The generalizability of our results to other countries is supported by our one-way deterministic sensitivity analysis, which highlights that treatment effectiveness, especially the PFS achieved by TARE, has the most significant impact on the ICER. Compared with costs, treatment effectiveness tends to be consistent across different countries. The choice between intention-to-treat or per-protocol analysis to determine the median PFS in TARE leads to significant variations in cost and effectiveness, resulting in divergent ICERs.

Recent trials have shown improved outcomes for systemic therapies, including immune checkpoint inhibitors, in the treatment of advanced HCC [23, 42, 43]. The Japanese guidelines recommend combination therapy with atezolizumab and bevacizumab as a first-line systemic therapy for advanced HCC not indicated for surgical resection, liver transplantation, percutaneous ablation, TACE, etc. [5]. Combination therapy with tremelimumab plus durvalumab was also added as a recommendation for first-line systemic therapy in a recent revision of the guideline in May 2023 [44]. However, regimens using immune checkpoint inhibitors are very expensive, and there are concerns about the impact on health economics. If TARE were to be approved in Japan, its benefit would be delaying the initiation of expensive systemic therapy by prolonging PFS compared with DEB-TACE, as shown in the state probability graph in our simulation (Fig. 2).

The method of cost estimation in this study is based on the Japanese healthcare system. However, since Japan’s health expenditure in relation to gross domestic product ranks fifth among the Organization for Economic Cooperation and Development countries and its population is aging more rapidly than that of other countries [45], the Japanese government’s policies toward high-cost procedures such as TARE may have implications for healthcare providers and policymakers in other countries that anticipate rising healthcare expenditures in the near future.

Our model assumed that continuous standard doses of atezolizumab + bevacizumab would be administered as systemic therapy in the TAE-refractory progressive state. This study may have overestimated the costs in the TAE-refractory progressive state because less expensive systemic pharmacotherapies may be preferred. However, even if less expensive pharmacotherapy is chosen in the TAE-refractory progressive state, our conclusion remains valid because our deterministic sensitivity analysis indicated that the ICER of TARE over DEB-TACE decreases as the cost of the TAE-refractory progressive state decreases.

Our findings have implications for determining reimbursement prices within the public health insurance system. The one-way deterministic sensitivity analysis in the primary analysis suggested that reducing the reimbursement price of radioactive microspheres from 1.440 million JPY (9,000 USD) to 1.399 million JPY (8,745 USD), approximately 2.8% lower than the price in the United Kingdom, would align the ICER with the willingness-to-pay threshold. In Japan, the reimbursement prices for expensive medical materials are officially determined for each category and are subject to biennial revisions. In 2019, cost-effectiveness analysis was introduced to determine the pricing of ultra-high-cost drugs and devices [13]. The price of radioactive microspheres used in TARE is remarkably high, necessitating rigorous negotiations based on cost-effectiveness analysis between the government and vendor. In this study, the price of radioactive microsphere, which is not yet approved in Japan, was set based on that in the United Kingdom, whereas in the deterministic sensitivity analysis (shown in Fig. 3), it was regarded as one of the variables. With this approach, the results of this study can contribute to a more evidence-based reimbursement policy. While estimating the costs for TARE, we assumed that the procedure fee was equivalent for DEB-TACE and TARE. However, in reality, TARE may be more expensive due to factors such as radiation protection. It may be worth considering policies such as setting a lower reimbursement price for radioactive microspheres and reallocating the saved funds to the procedure fee, which may benefit physicians and hospitals. However, this is a political issue involving the government, hospitals, physicians, and vendors, which is beyond the scope of this study.

This study has some limitations. First, the RCT data used to establish the transition probabilities in the model allowed for other local or systemic chemotherapies following the trial both in the intention-to-treat and per-protocol analyses [6]. Therefore, survival curves may incorporate the effects of treatments other than first-line TARE or DEB-TACE. Second, in this study, the costs of TARE were estimated assuming that it shared identical costs with DEB-TACE as TARE lacks Japanese health insurance approval. After TARE is approved in Japan, studies should be continued with real-world cost data on TARE. Third, we omitted percutaneous ablation or hepatic resection after TARE/DEB-TACE and liver transplantation to simplify the model. Fourth, DEB-TACE was used as the comparator in this study, although cTACE is employed more commonly than DEB-TACE for transarterial therapy for HCC in Japan. After TARE is introduced in Japan in the future, it will be necessary to perform a cost-effectiveness analysis using further research, once evidence on the comparative effectiveness of TARE and cTACE is established. Fifth, to estimate the potential costs for early severe complications, we assumed that there was no significant difference in the risk of severe complications between TARE and DEB-TACE according to the results of a previous RCT; however, due to the lack of late complications of TARE and DEB-TACE, late complications are not reflected in our simulation. In addition, we did not assess the impact of treatment-related adverse events on reducing quality-of-life estimates. We assume that the reduction in quality-of-life owing to complications does not last for more than a few months.

In conclusion, our study highlights that under specific conditions, TARE can be a more cost-effective treatment than DEB-TACE for unresectable HCC. The results of the primary analysis suggest that setting the reimbursement price of radioactive microspheres below 1.399 million JPY (8,745 USD), approximately 2.8% lower than the price in the United Kingdom, would allow TARE to be cost-effective. These findings have implications for evidence-based healthcare reimbursement policies and pricing negotiations and offer valuable insights into the complex cost-effectiveness landscape in primary liver-cancer treatment.

## Appendix E1: Details of the calculation formulas for transition probabilities

Based on the assumption that the survival curves of previous trials can be approximated by an exponential function, the transition probabilities were calculated using the following formula based on a previous study, which provides the equations: P (transition probabilities) = 1 − (0.5)^(length of cycle/median time to event)^ [27]. In the previous study, the model was relatively simple, consisting of only three states: “Progression-Free Survival,” “Progression State,” and “Death.” In contrast, in the present study, our model is more complex, as it considers the development of decompensated cirrhosis with certain transition probabilities. Therefore, it was necessary to provide a supplementary adjustment to the equation mentioned above.

Local to local (P1)

(1－(P_DecomTAE_/100))^Cy/12^×0.5^Cy/PFS^

* (1－(P_DecomTAE_/100))^Cy/12^ is the probability of not developing decompensated cirrhosis among survived cases after one cycle

Local to TAE-eligible progressive (P2)

(1－(P_DecomTAE_/100))^Cy/12^×(0.5^Cy/OS^－0.5^Cy/PFS^)

Local to decompensated cirrhosis (P3)

(1－(1－(P_DecomTAE_/100))^Cy/12^)×0.5^Cy/OS^

Local to death (P4)

1－0.5^Cy/OS^

TAE-refractory progressive to TAE-refractory progressive (P5)

(1－(P_DecomAtez_/100))^Cy/12^×0.5^Cy/OS^

TAE-refractory progressive to decompensated cirrhosis (P6)

(1－(1－(P_DecomAtez_/100))^Cy/12^))×0.5^Cy/OS^

TAE-refractory progressive to death (P7)

1－0.5^Cy/OS^

Decompensated cirrhosis to decompensated cirrhosis (P8)

P_SurvivalCirrh_^Cy/12^

Decompensated cirrhosis to death (P9)

1－P_SurvivalCirrh_^Cy/12^

Note- The probabilities represented by P1 to P9 each correspond to the probabilities in Fig. 1 and Table 2.

**Table:**

P_DecomTAE_ = Annual decompensation rate of TAE TAE-eligible population based on a previous cohort study [24] (%)			
P_DecomAtez_ = Annual decompensation of atezolizumab+bevacizumab therapy based on a previous cohort study [25] (%)			
P_SurvivalCirrh_ = Annual survival rate of decompensated cirrhosis based on a previous cohort study [26] (%)			
Cy = length of cycle (month)			
OS = median overall survival of TARE/DEB-TACE [6] or atezolizumab+bevacizumab therapy [23] (months)			
PFS = median progression-free survival after TARE/DEB-TACE [6] (months)			
DEB-TACE, transarterial chemoembolization with drug-eluting beads; TARE, transarterial radioembolization			
